# The Treatment of Narrow Nasal Passages

**Published:** 1916-01

**Authors:** A. Lohman


### THE TREATMENT OF NARROW NASAL PASSAGES.

BY DR. A. LOHMAN.

It is interesting at this date to note that in 1886, when the rhinologist, Dr. Eysell, speaking in Berlin at the general meeting of scientific investigators, threw out a suggestion as to the possibility of influencing the inner conformation of the nose by widening the upper jaw, he was met with a complete scepticism, and his suggestion soon forgotten. In 1900 Schroder-Benseler undertook the immense task of ascertaining the nature of the relationship between normal and abnormal jaw structure and the condition of the nasal passages. He examined and measured, in the various institutions and museums of Germany and Austria, altogether some twelve thousand skulls. These statistics he arranged under various classifications, type of skull, anatomical measurements, etc. But first and foremost he has placed the relationship between abnormal positions of the teeth and jaws and the condition of the nasal passages. From this research, which for the purpose in hand may well be considered exhaustive, two facts emerge beyond a shadow of doubt. First, that the jaw reaches its full breadth before the conclusion of the fourth year of life and does not afterwards change; secondly, that wide nasal passages are found only where there is a certain breadth of jaw, as measured across the hard palate. In other words, free nasal breathing is dependent upon the conformation of the jaw.

The importance of a free and proper use of the breathing apparatus can not be over-accentuated. Easy breathing is absolutely essential to the healthy development of a child's body. Where there is mouth-breathing or where the passage of air through the nose is insufficient, a healthy development of the chest, lungs and heart is impossible; speech, hearing and mental development are also injuriously affected. Already in 1905 rhinologists were considering the question of the treatment of mouth-breathing and constricted air passages by expansion of the jaw according to Dr. Schroder's method; and it ought at this date to be publicly recognized that one of the duties of the teacher is the immediate notification of the medical authorities of any case of abnormal breathing. The general development of a child is affected not only directly by improper breathing, but also indirectly through the digestive apparatus. The child who must breathe through its mouth while it eats is a pitiful case. The mastication of its food is necessarily imperfect and the food reaches the stomach improperly reduced and over-aerated. The blood of a mouth-breather is never thoroughly oxidated and impurities normally retained in the nostrils go straight to the lungs in a current of crude, unfiltered, unwarmed air. Last, but by no means least, in the list of evils attributable to mouth-breathing, is the effect upon the night's rest. The child, even if not actually wakened, is incessantly disturbed by the dryness of its mouth. Its sleep is always imperfect. The resultant effect upon the nervous system is incalculable. Mouth-breathers are slow of apprehension, incapable of attention, concentration or prolonged effort and often slightly deaf. School life is embittered for them by the efforts made on the part of uninstructed teachers to rouse them to efficiency, efforts which too often take the form of scoldings, threats and reproaches.

Dr. Schroder-Benseler's method of widening the jaws of mouth-breathers is the most important and most valuable item in modern orthodontia. He uses an upper expansion arch with a central screw, and usually fixes his apparatus to three teeth on either side of the jaw by means of gold caps. The wings of the arch are semi-circular and each sends out three arms to which the caps are fixed. The arch and its branches are from 2-3 mm. short of contact with the palate. The screw all but touches the roof of the mouth. If the screw be not placed as high as possible, dangerous pressure is exerted on the necks of the teeth and the result on the gums and the nasal floor is nil. Incidentally it is useful in some cases where the arch to begin with is extremely narrow, to use a series of screws, beginning with a very small one and gradually increasing the size. The business of widening the nasal passages must take precedence of any regulation of the teeth. The Schroder-Benseler plate is worn only at night. As soon as the expansion of the upper jaw is complete that of the lower must be taken in hand in order to restore normal occlusion. It is a safer orthodontic procedure to expand both arches at the same time.—*Ed. Register.*—Extracted from original paper in Archives for Zahnheilkunde by *The Dental Record.*

